# Validation of a predictive calculator for optimal glycemic control and time-in-tight-range following CGM sensor placement in type 1 diabetes and pancreatic diabetes: a prospective study

**DOI:** 10.1007/s12020-025-04385-7

**Published:** 2025-08-26

**Authors:** Fernando Sebastian-Valles, Juan Javier López-Hidalgo, Silvia Cañas Sierra, Victor Navas-Moreno, Jose Alfonso Arranz Martín, Miguel Antonio Sampedro-Núñez, Mónica Marazuela

**Affiliations:** Endocrinology and Nutrition Department, Hospital Universitario de La Princesa Instituto de Investigación Sanitaria de La Princesa, Universidad Autónoma de Madrid, Madrid, Spain

**Keywords:** Type 1 diabetes, Time in tight range, Continuous glucose monitoring, Socioeconomic status

## Abstract

**Background:**

Continuous glucose monitoring (CGM) has improved diabetes management, yet not all patients benefit equally. We previously developed a predictive calculator using clinical and socioeconomic variables to estimate the likelihood of achieving optimal control after CGM initiation. This study prospectively validated the calculator in a real-world cohort.

**Methods:**

A single-center prospective study included 102 adults with type 1 or pancreatic diabetes using multiple daily insulin injections, followed for three months. Optimal control was defined as time in range (TIR, 70–180 mg/dL) > 70% and time below range (TBR, <70 mg/dL) < 4%. Model performance was assessed using ROC analysis and correlation tests.

**Results:**

Of 102 participants, 85 completed follow-up (median age: 53.6 years; 48% women; median diabetes duration: 12.9 years; baseline HbA1c: 7.6%). Thirty-three (38.8%) achieved optimal control. The calculator showed moderate discrimination (AUC = 0.639) and significant correlations with TIR (*p* = 0.230, *p* = 0.023) and time in tight range (TITR, 70–140 mg/dL) (*p* = 0.271, *p* = 0.019). Overall accuracy was 61.9%, lower than in the original cohort. Smoking predicted non-completion (*p* = 0.038).

**Conclusions:**

The calculator shows moderate accuracy in predicting glycemic control and TITR after CGM initiation. CGM adherence remains a challenge, warranting further study in publicly funded healthcare settings.

## Introduction

Diabetes mellitus is one of the most prevalent chronic diseases worldwide and represents a major public health concern. In 2021, approximately 537 million adults were living with diabetes, and this number was predicted to continue rising in the coming decades [1]. Achieving optimal glycemic control is crucial to reducing diabetes-related morbidity and mortality, as chronic hyperglycemia is associated with an increased risk of both microvascular and macrovascular complications [2]. Several studies have demonstrated that sustained reductions in glycated hemoglobin (HbA1c) significantly lower the risk of diabetes complications [3].

Continuous glucose monitoring (CGM) has revolutionized diabetes management by providing real-time, detailed insights into interstitial glucose levels, thereby facilitating personalized treatment strategies and therapeutic optimization [4]. Its clinical benefits are well established across a wide range of diabetes populations and care settings CGM in diabetes management [5–11]. Unlike HbA1c, which reflects an average glucose level over the previous 2–3 months but does not capture glycemic variability or the occurrence of hypo- or hyperglycemic events, CGM-derived metrics allow for the evaluation of glucose patterns throughout day and night [12, 13]. Among these metrics, time in range (TIR, 70–180 mg/dL) has emerged as a key indicator of glycemic control, with studies supporting its association with long-term complication risk [14, 15]. A definition of optimal glycemic control based on these metrics (TIR exceeding 70% while maintaining time below range [TBR, < 70 mg/dL] below 4%) provides a more precise assessment of glycemic control without increasing the risk of hypoglycemia [16]. The use of composite these CGM-derived outcomes that integrate multiple metrics has been adopted in several studies to better reflect overall glycemic status [7, 17–19]. In addition, time in tight range (TITR,70–140 mg/dL) has emerged as a glucose metric relevant for both glycemic control and the prevention of complications [20–24].

Despite advances in diabetes technology, not all patients achieve optimal glycemic control with CGM, highlighting the importance of identifying predictors of response to this strategy. Recently, our group developed a predictive calculator based on clinical and socioeconomic variables to estimate the likelihood of achieving optimal glycemic control in a multicenter cohort of individuals with type 1 diabetes (T1D) or pancreatic diabetes [25]. This tool has the potential to enhance treatment individualization and optimize CGM use by identifying patients most likely to benefit from this technology.

The present study aimed to prospectively validate this predictive calculator in a cohort of individuals with T1D or pancreatic diabetes initiating CGM. The predictive ability of the calculator for optimal glycemic control (TIR > 70% and TBR < 4%) was determined after a 3-month follow-up period. This validation allowed assessing the applicability of the calculator in clinical practice and its potential use as a decision-support tool in diabetes management.

## Methods

### Study design

This is a single-center prospective study conducted between February 2024 and February 2025. The study included individuals with T1D or pancreatic diabetes (associated with chronic pancreatitis, exocrine pancreatic insufficiency, or pancreatectomy) who were receiving multiple daily insulin injections. All patients with pancreatic diabetes were treated with multiple daily insulin injections at study inclusion. In our setting, these individuals have access to public funding for continuous glucose monitoring (CGM) sensors. The primary objective was to prospectively validate the risk calculator previously developed by our group [25], with a follow-up period of three months. Adults using FreeStyle Libre 2 (Abbott) or Dexcom One (DexCom, Inc.) glucose sensors in routine clinical practice were enrolled. Exclusion criteria included individuals with type 2 diabetes mellitus, those lost to follow-up, and those with sensor usage below 70%, in accordance with international guidelines [13].

### Procedures

Prior to initiating CGM device use, all participants received a structured training session on device operation, in accordance with international recommendations [20]. The system employed consists of an electrochemical glucose oxidase-based sensor, which is implanted subcutaneously and replaced every 10 or 14 days, depending on the model. Interstitial glucose data were transmitted wirelessly to a receiver and stored in the cloud using the Libreview platform (for FreeStyle Libre 2) or Clarity (for Dexcom One).

All participants were provided with written instructions on using CGM data for real-time insulin dose adjustments and retrospective glucose data review to optimize insulin therapy. They were advised to modify insulin doses and hypoglycemia management based on glucose trends. Additionally, participants were informed that their glucose metrics would be downloaded three months after device placement and were instructed to attend regular follow-ups to review glucose control and report any issues.

### Data collection

Sociodemographic and clinical data, including laboratory tests and diabetes pharmacotherapy, were obtained through participant interviews and electronic health records review. Glucometrics data were retrieved from cloud platforms three months after sensor placement. The following variables were recorded: TIR (70–180 mg/dL), TITR (70–140 mg/dL), time below and above range (<70 mg/dL, >180 mg/dL, and >250 mg/dL, respectively), sensor usage, and coefficient of variation (CV). Additional sociodemographic and clinical data collected included sex, age, diabetes duration, body mass index (BMI), smoking status, most recent HbA1c value, CGM usage duration, disease duration, and insulin dose. HbA1c was measured using high-performance liquid chromatography (ADAMS A1c HA8180 V, ARKRAY®).Optimal control was defined as (TIR > 70% and time below range <70 mg/dL <4%).

All participants were informed about the study objectives and provided written informed consent, authorizing the use of their clinical data for research purposes. The study was approved by the Research Ethics Committee of Hospital de La Princesa, Madrid (Study Number: 2023-5401 4997-21/23).

Socioeconomic status (SES) was assessed using the mean net income per person as a proxy indicator of deprivation index [19, 26]. This information was not obtained from individual incomes; instead, we used the mean annual net income per person of the census tract for each individual, as periodically published by the National Institute of Statistics (2020); (National Institute of Statistics of Spain. (2021). Atlas de Distribución de Renta de los Hogares 2020. Retrieved from https://www.ine.es/componentes_inebase/ADRH_total_nacional.htm Accessed 1 February 2024).

### Sample size calculation

The sample size was estimated assuming that approximately 20% of the study population would achieve optimal glycemic control [25]. A minimum precision of 10% in proportion estimation was set based on its clinical relevance, and an attrition rate of 20% was assumed, consistent with previous diabetes studies [27]. Considering an alpha error of 5% and a power of 80%, the required sample size was estimated at 102 patients, who were consecutively recruited. The originally approved study protocol is available in the Supplementary Material S1.

### Statistical analysis

The normality of data distribution was assessed using the Kolmogorov-Smirnov test and graphical methods (normal probability plot). Continuous variables are presented as mean and standard deviation (SD) or median and interquartile range (IQR), while categorical variables are reported as absolute numbers and percentages.

The probability of achieving optimal glycemic control was estimated for each participant using a linear combination of coefficients derived from the original predictive calculator [25]. A receiver operating characteristic (ROC) curve analysis was performed to evaluate the predictive performance of the model for optimal glycemic control (TIR > 70% and time below range <70 mg/dL <4%). Two probability cut-off points were identified based on locally estimated scatterplot smoothing (LOESS) analysis and validated using logistic regression. Finally, the association between predicted probability and TITR and TIR was analyzed using Spearman’s Rho correlation.

Statistical analysis was performed using Stata 17.0 BE-Basic Edition (Lakeway Drive, College Station, TX, USA). Statistical significance was set at *p* < 0.05.

## Results

This study included 102 subjects treated with multiple daily insulin injections before the placement of a CGM sensor. The median age was 53.6 years (IQR: 39.4–66.6), and 49 individuals were women (48%). The median disease duration was 12.9 years (3.6–23.6), and HbA1c prior to sensor placement was 7.6% (6.9–8.8). Regarding diabetes type, 87 subjects (85.3%) had T1D, while 15 (14.7%) had pancreatic diabetes. The FreeStyle Libre 2 sensor was used in 83 cases (82.2%), and the Dexcom One sensor in 18 cases (17.8%). Baseline characteristics of the study population are detailed in Table 1.

**Table 1:** Baseline characteristics of the initial sample

Variables	Initial cohort	Final sample
	*N* = 102	*N* = 85
Age (years)	53.6 (39.4–66.6)	54.5 (41.2–67.0)
Sex (Woman)	49 (48.0%)	42 (49.4%)
Duration of diabetes (years)	12.9 (3.6–23.6)	10.9 (3.6–23.9)
HbA1c (%)	7.6 (6.9–8.8)	7.6 (6.9–8.5)
Insulin (IU/kg/day)	0.513 (0.362–0.667)	0.505 (0.362–0.658)
Type 1 Diabetes^a^	87 (85.3%)	73 (85.9%)
Pancreatic Diabetes	15 (14.7%)	12 (14.1%)
Annual income (€)	21,336 (14,339–23,866)	21,336 (14,389–24,468)
**BMI Classification**		
BMI < 18.5 Kg/m2	3 (2.9%)	2 (2.4%)
BMI 18.5–25 Kg/m2	50 (49.0%)	38 (44.7%)
BMI > 25 kg/m2	49 (48.0%)	45 (52.9%)
**Smoking Habit**		
Never Smoker	58 (56.9%)	50 (58.8%)
Smoker	17(16.7%)	11(14.1%)
Former Smoker	27 (26.5)	23(27.1)

Quantitative variables are expressed as median (interquartile range)

*HbA1c* glycated hemoglobin, *BMI* body mass index

^a^Three patients initially classified as type 1 diabetes were later reclassified as type 2 diabetes

**Fig. 1:**
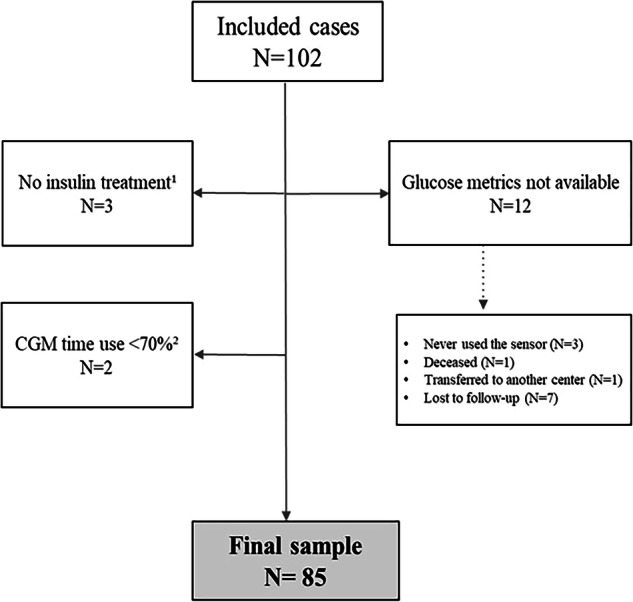
Study flowchart. ^1^ Three subjects were initially diagnosed with type 1 diabetes but were later reclassified as type 2 diabetes, leading to insulin withdrawal during follow-up. ^2^ Two subjects reported technical issues, precluding the collection of sufficient data for analysis at the end of follow-up. CGM continuous glucose monitoring.

Among the 102 subjects included in the study, 3 were reclassified as type 2 diabetes after diagnostic reassessment, 2 were unable to provide CGM data due to technical issues, and 12 had no available follow-up data at 3 months. Of these, 3 remained under follow-up but discontinued sensor use after the initial placement, 1 died due to metastatic urothelial cancer, 1 was transferred to another center, and 7 did not attend scheduled follow-up visits with medical staff or diabetes nurses. The final study sample comprised 85 participants (see Table 1). The study flowchart is presented in Fig. 1.

### Inappropriate device use

We analyzed factors associated with inadequate CGM use in the 10 subjects with unjustified missing data compared to the final sample of 85 participants. The only significant difference observed was smoking status, with a higher proportion of smokers among those with missing data (40% vs. 8.2% in the adherent group, *p* = 0.039). No statistically significant differences were found in other variables between users and non-users: age (53.84 ± 17.5 years vs. 53.5 ± 15.8 years, *p* = 0.956), disease duration (15.6 ± 14.4 vs. 16.4 ± 15.4 years, *p* = 0.854), HbA1c (7.9 ± 1.6 vs. 8.1 ± 1.6, *p* = 0.714), insulin dose (0.547 ± 0.265 vs. 0.583 ± 0.156, *p* = 0.677), and per capita net income (€20,580 ± 6,625 vs. €20,266 ± 5,631, *p* = 0.8861). A trend toward lower adherence was observed in underweight (33%) and normal-weight subjects (18.4%) compared to overweight individuals (4.4%), although the difference was not statistically significant (*p* = 0.089).

### Glucose metrics

Among the 85 subjects who completed follow-up, median (IQR) values were obtained for the following glucose metrics: TIR (70–180 mg/dL), 72% (52–86);TBR ( < 70 mg/dL); 1% (0–3); glucose management indicator (GMI); 7.1% (6.5–7.7); TITR (70–140 mg/dL), 43% (28–59).

A total of 33 subjects (38.8%) achieved optimal glycemic control after sensor placement. Glucose metrics are detailed in Table 2.

**Table 2:** Glucose metrics

Variables	Obs
	*N* = 85
Glucose average (mg/dL)	157 (133.5–184.5)
Time in range (70–180 mg/dL)	72 (52–86)
Time in tight range (70–140 mg/dL)^a^	43 (28–59)
Time below range (<70 mg/dL)	1 (0–3)
Optimal control (%)	33 (38.8%)
CGM time use (%)	96 (94–98)
Time above range (>180 mg/dL)	22 (11–32)
Time above range (>250 mg/dL)	5 (0–16)
Coefficient of variation	31.9 (27.4–35.5)
GMI (%)	7.1 (6.5–7.7)

Quantitative variables are expressed as median (interquartile range)

*CGM* continuous glucose monitoring, *GMI* glucose management indicator

^a^75 participants

### Application of the predictive calculator

**Fig. 2:**
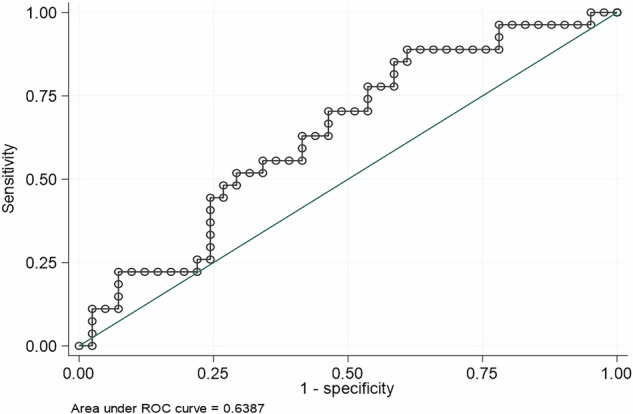
ROC curve for optimal control based on the predicted probability prior to sensor placement. The figure shows a moderate discriminative ability of the predictive calculator. Optimal control: Time in range (TIR, 70–180 mg/dL) >70% and time below range (TBR, <70 mg/dL) <4%. AUC area under the curve, ROC receiver operating characteristic curve.

Non-parametric ROC curves were constructed to evaluate the discriminatory capacity of the predicted probability of achieving optimal glycemic control obtained with the predictive calculator. ROC curve analysis yielded an area under the curve (AUC) of 0.639, which indicated moderate discriminative ability (Fig. 2).

**Fig. 3:**
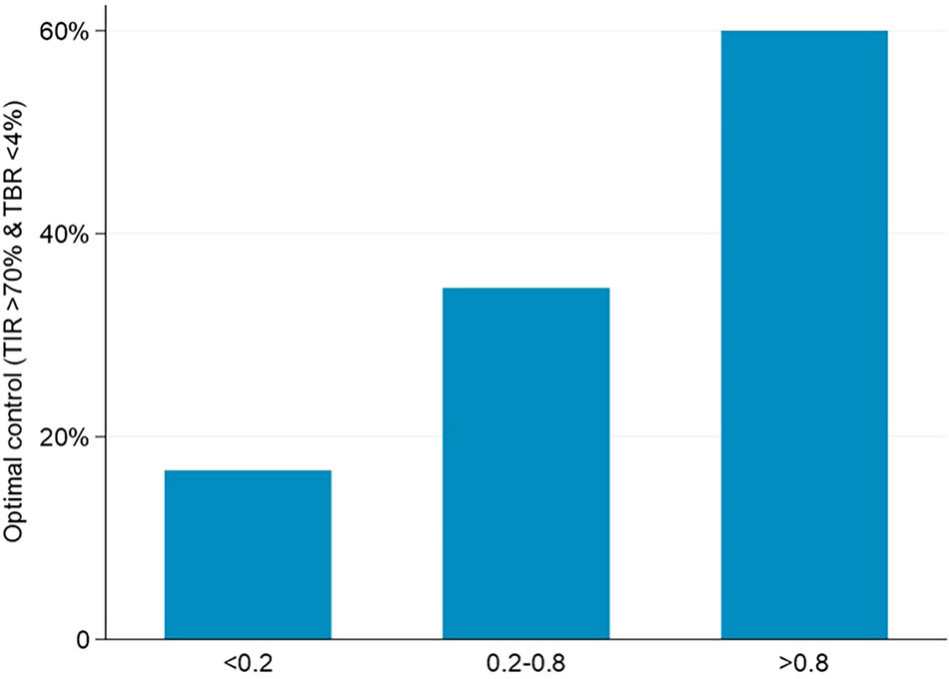
Relationship between predicted probability and the proportion of subjects achieving optimal glycemic control after sensor placement. Proportions of patients achieving optimal glycemic control according to their predicted probability. The graph illustrates the proportion of patients achieving optimal glycemic control (TIR 70–180 mg/dL >70% and TBR <70 mg/dL <4%) in different groups defined by two cut-off points for the predictive probability obtained with the calculator: Probability <0.2, probability (0.2–0.8), and probability >0.8.

To define probability cut-off points for glycemic control, a LOESS curve was applied (Supplementary Material S2), identifying two cut-offs (0.2 and 0.8) that allowed establishing three probability categories : Probability <0.2, optimal control was achieved in 16.7% of the cases in the cohort; probability 0.2–0.8, in which optimal control was achieved in 34.6% of the cases; and probability >0.8, in which optimal control was achieved in 60% of the cases (Fig. 3).

The logistic regression model showed a statistically significant association between the predicted probability obtained with the calculator and achievement of optimal control (OR 2.919 [95% CI: 1.05–8.12], *p* = 0.040). The model showed a higher capacity to identify subjects without glycemic control (specificity 96.1%) compared to well-controlled individuals (sensitivity 9.1%), with a positive predictive value of 60.0%, a negative predictive value of 62.3%, and an overall classification accuracy of 61.9%.

**Fig. 4:**
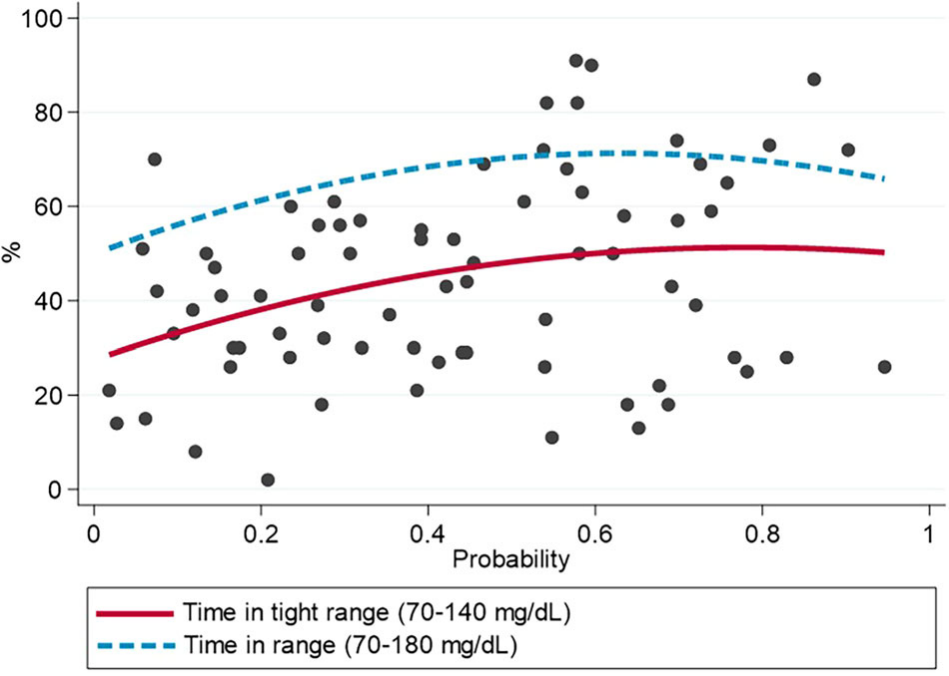
Association between the predicted probability obtained with the calculator and time in tight range and time in range. The scatter plot represents the Spearman correlation analysis for the association between time in tight range (70–140 mg/dL) and the probability of achieving optimal glycemic control (TIR 70–180 mg/dL >70% and TBR <70 mg/dL <4%) obtained with the predictive calculator. The adjusting curve is shown in red. The dashed blue line represents the adjusting curve for similar analysis for conventional time in range (70–180 mg/dL). A moderate association is observed for both variables.

Finally, we analyzed the association between the probability predicted by the calculator and the novel metric TITR (70–140 mg/dL). A significant positive correlation was observed (Spearman’s Rho = 0.271, *p* = 0.019). A statistically significant association with conventional TIR was also identified, although the association was slightly weaker in this case (Spearman’s Rho = 0.230, *p* = 0.023) (Fig. 4).

## Discussion

This study aimed to prospectively validate a predictive calculator based on clinical and socioeconomic variables commonly used in clinical practice. The calculator estimates the probability of achieving optimal glycemic control, defined as TIR > 70% and TBR < 4%, in individuals with T1D or pancreatic diabetes after initiating CGM in a healthcare system with unrestricted access to this technology. Our findings indicate that the predictions made by the calculator are moderately associated with actual optimal glycemic control and TITR.

Our analysis indicates that the predictive calculator demonstrates moderate discriminative capacity, particularly in identifying individuals with poor glycemic control. Although it was significantly associated with both TIR and TITR, its overall performance was reduced compared to the original cohort, with a decline in the AUC from 0.736–0.639. This suggests a reduced capacity to predict optimal glycemic control. Several factors may explain this decrease in predictive ability. First, glycemia is a variable highly influenced by biological [28], psychological [29] and social factors [19, 30], which are not always easily quantifiable and may introduce variability into predictive models. Second, the validation cohort differed significantly from the original sample in several key aspects, including older age (53 vs. 47 years), shorter disease duration (12 vs. 21 years), and a 20% higher average income (€21,336 vs. €16,948) [25]. These differences may have contributed to the higher proportion of individuals achieving optimal glycemic control in the validation cohort (38.8% vs. 21%), potentially affecting the generalizability of the model. Moreover, increased expertise among healthcare professionals in managing CGM over time could have played a role in these findings. The sample size (102 participants) may have limited the ability to detect statistically significant associations. However, we believe that using a cohort representative of the actual patient population receiving CGM devices in a tertiary care center enhances the ecological validity of the study.

Additionally, our findings provide insights into CGM adherence and utilization in a publicly funded healthcare system with unrestricted access to the technology. We observed that 16% of participants had no available data at the three-month follow-up, with 9.8% lost to follow-up without justification or voluntarily discontinuing the device. While no clear determinants of CGM discontinuation were identified, a trend toward lower adherence among smokers was noted, aligning with previous studies linking smoking to poorer glycemic outcomes in diabetes [31, 32] and a reduced benefit from CGM use in T1D [33]. However, due to the follow-up losses, it was not possible to establish causality. Improving CGM adherence may require structured and ongoing diabetes education programs [34], which have demonstrated efficacy in optimizing self-management and device utilization [35–37]. Our protocol included an initial training session; however, no additional educational interventions were implemented, which may have influenced discontinuation rates. Further research is needed to identify the factors contributing to suboptimal CGM utilization and to ensure the sustainability of healthcare resources.

This predictive tool may support individualized decision-making in real-world settings by identifying individuals at higher risk of suboptimal response to CGM. In practice, this could help prioritize targeted educational or behavioral interventions to improve sensor use and optimize outcomes—particularly in publicly funded systems where CGM access is guaranteed, but adherence and benefit vary. Nonetheless, given the reduced discriminative performance in the validation cohort and the clinical differences from the original population, these findings should be interpreted with caution. Future refinement of the calculator should incorporate behavioral, psychosocial, and potentially device-specific factors to enhance predictive accuracy and ensure applicability across diverse clinical contexts.

This study has several limitations. First, to reflect real-world clinical practice, we included two different CGM sensor brands, which may have introduced variability in glycemic measurements. Although both devices are widely used and clinically validated, differences in features such as alarm functionality and data visualization may influence user engagement and behavior. Given the limited sample size, we were unable to perform adequately powered subgroup analyses by sensor type. Additionally, key variables relevant to glycemic control and smoking, such as dietary habits [38, 39], physical activity [40, 41], personal social situation [42, 43] or mental health history [29, 44], were not considered, limiting the analysis to clinical and socioeconomic factors. Furthermore, the use of census-section net income as a proxy for SES does not reflect individual-level SES, but rather the SES of a given geographical area. Ideally, hierarchical models accounting for both individual and aggregate census-level variables should have been used. Nonetheless, multiple studies [45–49] support the consideration of census-based SES variables as proxies for individual SES. Finally, since the study aimed to evaluate the probability of achieving glycemic control based on glucose metrics immediately after CGM initiation, no follow-up HbA1c measurements were obtained.

In conclusion, the predictive calculator based on clinical and socioeconomic variables demonstrated moderate accuracy in estimating the likelihood of achieving optimal glycemic control and TITR in individuals with T1D or pancreatic diabetes treated with multiple daily injections, particularly in identifying patients at risk of poor glycemic control. However, a substantial proportion of patients did not use CGM devices effectively following initiation. This, combined with the high attrition rate and limited sample size, may reduce the external validity of our findings. Further studies are necessary to better identify the behavioral patterns and determinants associated with suboptimal CGM utilization in healthcare systems with unrestricted access to this technology.

## Supplementary information

Supplementary S1

Supplementary S2

## Data Availability

The dataset supporting the findings of this study includes new, unpublished data. These data are available from the corresponding authors upon reasonable request.
