# Supplementary material for: Validation of a predictive calculator for optimal glycemic control and time-in-tight-range following CGM sensor placement in type 1 diabetes and pancreatic diabetes: a prospective study
Source: Endocrine. 2025 Aug 26;90(2):660–8. doi: 10.1007/s12020-025-04385-7 (PMC12572046; doi:10.1007/s12020-025-04385-7)
Supplement: Supplementary file 1 — Supplementary S1 [file 12020_2025_4385_MOESM1_ESM.pdf]

VALIDACIÓN EXTERNA DE UNA CALCULADORA DE RIESGO QUE PREDIGA LA PROBABILIDAD DE CONTROL ÓPTIMO EN USUARIOS DE MONITORIZACIÓN CONTINUA DE GLUCOSA

VERSIÓN 1.1 06-12-2023

INVESTIGADOR PRINCIPAL:

Fernando Sebastián Valles (servicio de Endocrinología)[1]

INVESTIGADORES COLABORADORES:

José Alfonso Arranz Martín (servicio de Endocrinología)[1]
Miguel Antonio Sampedro Núñez (servicio de Endocrinología)[1]
Mónica Marazuela Azpiroz (servicio de Endocrinología)[1]

1. Servicio de Endocrinología y Nutrición.
Hospital Universitario de La Princesa. Madrid

Madrid, 09 de noviembre de 2023.

INTRODUCCIÓN:

La Diabetes Mellitus tipo 1 es una enfermedad en la que todos los pacientes reciben tratamiento con insulina para controlar sus niveles glucémicos. Para recibir la dosis adecuada de insulina, se requiere una medición precisa de la glucosa en sangre con un medidor de glucosa por punción digital. La evidencia que respalda que un control estricto de la glucemia puede mitigar los efectos adversos de la hiperglucemia en pacientes con diabetes tipo 1 (DT1) ha sido sólida durante años (1-3) El autocontrol de los niveles de glucosa capilar marcó el inicio de un mejor control crónico mediante la monitorización de la glucosa (4-5). Durante la última década, la llegada de los dispositivos de monitorización continua de glucosa (MCG) se ha asociado con una reducción de los niveles de HbA1c tanto en niños como en adultos con diabetes tratada con insulina (6-10). Además, la adopción generalizada de estos dispositivos ha logrado mejorar el control glucémico en estudios de datos del mundo real con grupos de pacientes muy diversos (11).

Los sensores MCG ofrecen un espectro de métricas de glucosa cuya importancia en la evaluación de la glucémica de personas con diabetes está respaldada por directrices de consenso internacional (12-13). Estas métricas de glucosa se correlacionan adecuadamente con los niveles de hemoglobina glicosilada (HbA1c) (14) y, en algunos casos, pueden ofrecer incluso más información (15). Actualmente, los estudios clínicos prospectivos emplean dispositivos MCG (12) para recopilar datos que complementan las observaciones de las intervenciones terapéuticas en la diabetes tipo 1. El porcentaje de tiempo en rango (TIR) entre 70 y 180 mg/dL es una de las métricas de glucosa derivadas de los sistemas CGM y se usa comúnmente como medida de resultado primaria en estudios de diabetes Tipo 1. Los cambios en el TIR del 5% se consideran clínicamente significativos (16) y cuando esta variable supera el 70% se considera indicativo de un objetivo de HbA1c (17).

Por otro lado, la hipoglucemia grave es una situación clínica que no se puede clasificar mediante métricas de MCG. Así, el tiempo por debajo del rango <70 mg/dL (TBR) se propone como el mejor predictor de hipoglucemia grave (18), y el umbral recomendado actualmente es TBR <4% (12). Dado que la hipoglucemia es un factor limitante en el manejo de la glucemia, están surgiendo estudios que abordan la predicción de eventos hipoglucémicos (19,20). Sin embargo, aún no se han desarrollado modelos predictivos que evalúen la probabilidad de lograr simultáneamente el control óptimo recomendado con TIR >70% y TBR <4% en el momento de la colocación del sensor MCG.

Resultados previos:
Para ello nos remitimos al estudio **6-10-22, acta CEIm 18/22** de cohortes retrospectivo cuyo objetivo secundario la elaboración de modelos predictivos de control glucémico en personas con diabetes tipo 1.

Este trabajo incluyó a 1072 pacientes con diabetes tipo 1 portadores de monitorización flash de glucosa procedentes de 2 centros españoles (H. U. La Princesa de Madrid, H.U. Severo Ochoa de Leganés).

En este trabajo se registraron parámetros clínicos como la duración de la enfermedad, hemoglobina glicada (HbA1c) previa a la colocación del sensor, presencia de complicaciones crónicas, dosis diaria de insulina/kg.

Adicionalmente, dado la evidencia creciente que están ganando los parámetros de medición del status socioeconómica (SES) y su gran impacto en la salud en general y la diabetes en particular (21-24), se optó por extraer la renta neta anual media por persona asociada a la sección censal de cada individuo que muestra la renta media en la unidad demográfica más pequeña que disponible en España como marcador del entorno socioeconómico del paciente.

Inicialmente se dividió la muestra total en dos partes de forma aleatoria para utilizar una muestra para la creación del modelo estadístico predictivo y otra para la validación externa del modelo construido en la primera. Por métodos computacionales se eligió el mejor modelo de regresión logística que incluyera las variables predictoras más relevantes para el outcome final (control óptimo). Se seleccionó el mejor modelo por medio del Criterio de Información de Akike (AIC) y se consideró necesario que el modelo ajustara por el estadístico de Pearson y de Hosmer-Lemeshow. Finalmente, se estudió la fiabilidad del modelo mediante validación externa en la cohorte de validación.

Los resultados mostraron que el modelo que constaba de las características predictoras más potentes incluyó la renta neta anual por persona (en miles de €), Odds Ratio (OR)= 1,045 p = 0,014, el sexo OR=1,44 p=0,800, la edad OR=1,032 p< 0,001; años de duración de la DM1 OR=0,965, P< 0,001), la HbA1c previa a FGM OR=0,838 p=0,213, las dosis insulina/kg OR 0,026; P < 0,001) y la interacción del sexo con la HbA1c (p=0.932). El modelo elegido presentaba en la cohorte de validación una especificidad del 72,6% una sensibilidad del 67,3%.

La calculadora de riesgo creada con el modelo matemático que incluye las variables mencionadas, predijo adecuadamente la presencia o ausencia de control óptimo en el 80,4% de los pacientes de la cohorte de validación.

Todos estos resultados fueron presentados por medio de una Comunicación Oral el 19 de octubre de 2023 en el Congreso Nacional de la Sociedad Española de Endocrinología y Nutrición.

En este contexto, en base al estudio mencionado se desea realizar una validación prospectiva del modelo predictivos basados en variables clínicas y socioeconómicas que muestra una precisión estimada del 80%.

El objetivo del presente trabajo es replicar el uso de la calculadora de riesgo de forma prospectiva.

HIPÓTESIS DE TRABAJO:
La hipótesis de partida del trabajo supone que la probabilidad de alcanzar el control óptimo (definido como tiempo en rango 70-180mg/dL >70% con tiempo por debajo de rango <70 mg/dl <4%) en métricas de glucosa de una persona con diabetes se puede calcular predictivamente con variables clínicas disponibles en el momento de la colocación del sensor.

OBJETIVOS:
Objetivo principal:

- Validación prospectiva de la calculadora de riesgo previamente desarrollada que estudia la probabilidad que tiene un individuo de estar dentro del control óptimo utilizando un sensor de monitorización continua de glucosa.

Objetivos secundarios:

- Establecer factores de riesgo clínicos y sociodemográficos asociados a un mal uso o uso insuficiente del sensor de monitorización continua de glucosa.

MATERIAL Y MÉTODOS:

1. DISEÑO

Se trata de un estudio de cohorte prospectivo en el que se calculará la probabilidad de alcanzar el control glucémico óptimo (Tiempo en rango >70% con tiempo por debajo de rango <4%) en el momento de colocar un sensor de monitorización continua de glucosa. Se reevaluarán las métricas de glucosa del sensor 3 meses tras su colocación para verificar la coherencia con las predicciones realizadas

Criterios de inclusión:
•Estar incluido en listado de formación para la colocación de sensor de Monitorización Continua de Glucosa FreesStyle 2 o Dexcom One a fecha de 1 de diciembre de 2023 con diagnóstico de Diabetes Mellitus tipo 1 (DM1), Diabetes Mellitus relacionada con la Fibrosis Quística (DMFQ) y Diabetes Mellitus pancreática (DM3c).
• Mantener seguimiento clínico habitual en la unidad que proporciona el sistema MCG.

Criterios de exclusión:

• Pacientes con diagnóstico de Diabetes Mellitus tipo 2.
• Pacientes con un uso del sensor menor del 70% en el momento de la descarga de datos (no criterio de exclusión para objetivo secundario).

2. VARIABLES DEL ESTUDIO

Se revisarán las historias clínicas de los pacientes incluidos para obtener las siguientes variables:

2.1. Variables demográficas

- Sexo.
- Fecha de nacimiento.
- Dirección postal.

2.2. Variables glucométricas en Monitorización Continua de Glucosa que se descargarán en informe de 14 días en el momento de la lectura de datos a los 3 meses de la colocación del dispositivo.

- Porcentaje de tiempo en rango (TER)
- Promedio de lecturas diarias de sensor.
- Porcentaje de tiempo de uso del sensor.
- Coeficiente de variación.
- Desviación estándar.
- Porcentaje de tiempo por debajo de rango <70 mg/dL.
- Porcentaje de tiempo en por encima de rango 180 mg/dL
- Porcentaje de tiempo en por encima de rango 250 mg/dL
- GMI (indicador de gestión de glucosa).

2.3 Variables clínicas

- Tipo de Diabetes Mellitus.
- Fecha de debut.
- Peso.
- Talla.
- Tabaco activo definido como el consumo de 1 cigarrillo al día o más.
- Dosis total de insulina diaria.
- Hemoglobina glicosilada (HbA1c) más reciente.
- Antecedentes de retinopatía diabética.
- Antecedentes de nefropatía diabética.

2.4 Variable socioeconómica

A través de la dirección postal de cada paciente se obtendrá la sección censal correspondiente y se cotejará con su renta neta anual media por persona que publica

periódicamente el Instituto Nacional de Estadística (Atlas de Distribución de Renta de los Hogares 2020. Recuperado de https://www.ine.es/componentes_inebase/ADRH_total_nacional.htm Consultado el 24 de octubre de 2023).

3. CRONOGRAMA DE TRABAJO

- Reclutamiento de 102 pacientes en los que es previsible la colocación de un sensor MCG por encontrarse en lista de formación en la Unidad de Diabetes.
- Firma de consentimiento informado para el acceso a Hª Clínica en el momento de la colocación del sensor.
- Recogida de datos clínicos tras la firma del consentimiento informado.
- Creación de una base de datos específica que recoja las variables clínicas y socioeconómicas
- Análisis estadístico de todos los datos recogidos tras 3 meses de uso del sensor.
- Escritura del artículo científico, publicación y envío a un congreso internacional.

4. MÉTODOS ESTADÍSTICOS:

4.1.Tamaño muestral

En la población de estudio es esperable que un 20% de los individuos se encuentren dentro del control glucémico óptimo. La precisión mínima en el cálculo de proporciones de la herramienta la establecemos en el 10% por su significado clínico y suponemos una tasa de abandono mínima del 20% como en otros trabajos en diabetes (25). Por lo cual, considerando un error alfa del 5% y una potencia del 80%, el tamaño muestral aproximado necesario para el estudio es de 102 pacientes.

Dado que el ritmo de colocación de sensores mensuales en los hospitales de la comunidad de Madrid varía entre 20-40 cada 3 meses, consideramos necesaria una duración del estudio de 9 meses.

4.2. Análisis estadístico

Se compararán la probabilidad predicha con las métricas de glucosa reales de cada paciente. Se realizará una tabla de riesgos pronósticos para evaluar la validad global de la calculadora de riesgo. Las variables cualitativas serán analizadas mediante chi-cuadrado. Las variables cuantitativas serán analizadas mediante la prueba t de student o la prueba de la U de Mann-Whitney según la distribución de la variable.

4.3. Calculadora de riesgo

La calculadora de riesgo se encuentra creada por medio del paquete estadístico STATA 17.0 BE-Basic Edition statistical software (Lakeway Drive, College Station, Texas, USA), licencia adquirida en 2021 en ordenadores del Hospital Universitario de La Princesa. La aplicación de la misma se lleva a cabo aplicando los valores de cada individuo a las 6 variables incluidas en la calculadora (sexo, edad, tiempo de evolución, dosis de insulina/kg/día, HbA1c y renta neta media). La calculadora aporta una probabilidad que varía entre 0 y 1. Los valores inferiores a 0,5 se considerarán negativos y los valores superiores a 0,5, positivos.

5. ASPECTOS ÉTICOS Y LEGALES

El tratamiento y la comunicación de los datos de carácter personal de todos los sujetos participantes se ajustarán a lo dispuesto en la Ley Orgánica 3/2018 y el Reglamento Europeo 2016/679 de Protección de Datos. El estudio se llevará a cabo de acuerdo con los requerimientos expresados en la Declaración de Helsinki (revisión de Fortaleza 2013) así como la legislación vigente relativa a la realización de estudios observacionales.

El acceso a historias clínicas y la recogida y manejo de datos confidenciales se realizará exclusivamente desde el sistema informático del Hospital Universitario de La Princesa y del Hospital Universitario Severo Ochoa, y no será necesario entrevistar a los pacientes. Se solicitará Consentimiento informado a los pacientes dado el carácter prospectivo del estudio. El proyecto planteado tiene un diseño de tipo observacional y no se aplica ningún tipo de intervención, ya sea diagnóstica o terapéutica fuera de la realizada dentro de la práctica clínica habitual. Se trata de la recogida de datos de la historia clínica de pacientes seleccionados en los que ya está asignada, por práctica habitual de la medicina, una estrategia terapéutica concreta, no existiendo, por lo tanto, ninguna posibilidad de interferencia con los hábitos de prescripción del médico ni de ocasionar ninguna alteración sobre los datos que constan en la historia clínica.

Los datos obtenidos se protegerán mediante la asignación a cada historia clínica de un número de registro que quedaría guardado en un fichero al que nadie ajeno al estudio o a su evaluación tendrá acceso, de modo que el tratamiento posterior de la información recogida en la hoja de datos se realiza de forma seudonimizada y se mantendrá en todo momento la confidencialidad. Al concluirse el período de la recogida de información, los documentos que cruzan los datos de identificación de los pacientes con el código asignado serán destruidos. Cualquiera de los datos con los que se vaya a trabajar no se utilizarán nunca con un fin distinto al del objetivo del estudio.

MEMORIA ECONÓMICA: se trata de un estudio en el que se
analizan parámetros clínicos y glucométricos de práctica habitual por lo que no supondrá ningún sobrecoste económico al centro.

DIFUSIÓN DE DATOS

Los resultados de este trabajo serán presentados en congresos nacionales e internacionales de Endocrinología. Igualmente serán remitidos en forma de artículo para su valoración en revistas de Endocrinología.

BIBLIOGRAFÍA

1. Lachin JM, Nathan DM, on behalf of the DCCT/EDIC Research Group. Understanding Metabolic Memory: The Prolonged Influence of Glycemia During the Diabetes Control and Complications Trial (DCCT) on Future Risks of Complications During

the Study of the Epidemiology of Diabetes Interventions and Complications (EDIC). Diabetes Care 2021;44(10):2216–2224; doi: 10.2337/dc20-3097.

2. Klein R. Hyperglycemie and Microvascular and Macrovascular Disease in Diabetes. Diabetes Care 1995;18(2):258–268; doi: 10.2337/diacare.18.2.258.

3. Wiseman MJ, Viberti G. Genesis and evolution of proteinuria in diabetes mellitus. Ricerca in clinica e in laboratorio 1985;15(1):9–16; doi: 10.1007/BF03029156.

4. Barnett AH. Diabetic Control and the Effect of Changing a Diabetic Clinic to Modern Management. Diabetic Medicine 1985;2(1):57–58; doi: 10.1111/j.1464-5491.1985.tb00594.x.

5. Sönksen PH, Judd S, Lowy C. Home Monitoring of Blood Glucose: New Approach to Management of Insulin-dependent Diabetic Patients in Great Britain. Diabetes Care 1980;3(1):100–107; doi: 10.2337/diacare.3.1.100.

6. Wong CA, Miller VA, Murphy K, et al. Effect of Financial Incentives on Glucose Monitoring Adherence and Glycemic Control Among Adolescents and Young Adults With Type 1 Diabetes: A Randomized Clinical Trial. JAMA Pediatrics 2017;171(12):1176–1183; doi: 10.1001/jamapediatrics.2017.3233.

7. Beck RW, Riddlesworth T, Ruedy K, et al. Effect of Continuous Glucose Monitoring on Glycemic Control in Adults With Type 1 Diabetes Using Insulin Injections: The DIAMOND Randomized Clinical Trial. JAMA 2017;317(4):371; doi: 10.1001/jama.2016.19975.

8. Aleppo G, Ruedy KJ, Riddlesworth TD, et al. REPLACE-BG: A Randomized Trial Comparing Continuous Glucose Monitoring With and Without Routine Blood Glucose Monitoring in Adults With Well-Controlled Type 1 Diabetes. Diabetes Care 2017;40(4):538–545; doi: 10.2337/dc16-2482.

9. Šoupal J, Petruželková L, Grunberger G, et al. Glycemic Outcomes in Adults With T1D Are Impacted More by Continuous Glucose Monitoring Than by Insulin Delivery Method: 3 Years of Follow-Up From the COMISAIR Study. Diabetes Care 2019;43(1):37–43; doi: 10.2337/dc19-0888.

10. Leelarathna L, Evans ML, Neupane S, et al. Intermittently Scanned Continuous Glucose Monitoring for Type 1 Diabetes. N Engl J Med 2022;387(16):1477–1487; doi: 10.1056/NEJMoa2205650.

11. Moreno-Fernandez J, Sastre J, Pinés P, et al. To evaluate the use and clinical effect of intermittently scanned continuous glucose monitoring in adults with type 1 diabetes: Results of a multicentre study. Endocrinología, Diabetes y Nutrición (English ed) 2023;70(4):270–276; doi: 10.1016/j.endien.2023.03.013.

12. Battelino T, Alexander CM, Amiel SA, et al. Continuous glucose monitoring and metrics for clinical trials: an international consensus statement. The Lancet Diabetes & Endocrinology 2023;11(1):42–57; doi: 10.1016/S2213-8587(22)00319-9.

13. Agiostratidou G, Anhalt H, Ball D, et al. Standardizing Clinically Meaningful Outcome Measures Beyond HbA1c for Type 1 Diabetes: A Consensus Report of the American Association of Clinical Endocrinologists, the American Association of Diabetes Educators, the American Diabetes Association, the Endocrine Society, JDRF International, The Leona M. and Harry B. Helmsley Charitable Trust, the Pediatric Endocrine Society, and the T1D Exchange. Diabetes Care 2017;40(12):1622–1630; doi: 10.2337/dc17-1624.

14. Xing D, Kollman C, Beck RW, et al. Optimal Sampling Intervals to Assess Long-Term Glycemic Control Using Continuous Glucose Monitoring. Diabetes Technology & Therapeutics 2011;13(3):351–358; doi: 10.1089/dia.2010.0156.

15. Beck RW, Connor CG, Mullen DM, et al. The Fallacy of Average: How Using HbA1c Alone to Assess Glycemic Control Can Be Misleading. Diabetes Care 2017;40(8):994–999; doi: 10.2337/dc17-0636.

16. Vigersky RA, McMahon C. The Relationship of Hemoglobin A1C to Time-in-Range in Patients with Diabetes. Diabetes Technology & Therapeutics 2019;21(2):81–85; doi: 10.1089/dia.2018.0310.

17. Beck RW, Bergenstal RM, Cheng P, et al. The Relationships Between Time in Range, Hyperglycemia Metrics, and HbA1c. J Diabetes Sci Technol 2019;13(4):614–626; doi: 10.1177/1932296818822496.

18. Hermanns N, Ehrmann D, Heinemann L, et al. Real-Time Continuous Glucose Monitoring Can Predict Severe Hypoglycemia in People with Type 1 Diabetes: Combined Analysis of the HypoDE and DIAMOND Trials. Diabetes Technology & Therapeutics 2022;24(9):603–610; doi: 10.1089/dia.2022.0130.

19. Li J, Ma X, Tobore I, et al. A Novel CGM Metric-Gradient and Combining Mean Sensor Glucose Enable to Improve the Prediction of Nocturnal Hypoglycemic Events in Patients with Diabetes. Kokkinos A. ed. Journal of Diabetes Research 2020;2020:8830774; doi: 10.1155/2020/8830774.

20. Georga EI, Protopappas VC, Ardigò D, et al. A Glucose Model Based on Support Vector Regression for the Prediction of Hypoglycemic Events Under Free-Living Conditions. Diabetes Technology & Therapeutics 2013;15(8):634–643; doi: 10.1089/dia.2012.0285.

21. Dover AR, Strachan MWJ, McKnight JA, et al. Socioeconomic deprivation, technology use, C-peptide, smoking and other predictors of glycaemic control in adults with type 1 diabetes. Diabetic Medicine 2021;38(3):e14445; doi: 10.1111/dme.14445.

22. Alvarez-Ramos P, Jimenez-Carmona S, Alemany-Marquez P, et al. Socioeconomic deprivation and development of diabetic retinopathy in patients with type 1 diabetes mellitus. BMJ Open Diab Res Care 2020;8(2):e001387; doi: 10.1136/bmjdrc-2020-001387.

23. Mönkemöller K, Müller-Godeffroy E, Lilienthal E, et al. The association between socio-economic status and diabetes care and outcome in children with diabetes type 1 in Germany: The DIAS study (diabetes and social disparities). Pediatr Diabetes 2019;pedi.12847; doi: 10.1111/pedi.12847.

24 . Rawshani A, Svensson A-M, Rosengren A, et al. Impact of Socioeconomic Status on Cardiovascular Disease and Mortality in 24,947 Individuals With Type 1 Diabetes. Diabetes Care 2015;38(8):1518–1527; doi: 10.2337/dc15-0145.

25. Fernando Gomez-Peralta, Cristina Abreu, Elsa Fernández-Rubio, Laura Cotovad, Pedro Pujante, Sonia Gaztambide, Diego Bellido, Edelmiro Menéndez Torre, Santiago Ruiz-Valdepeñas, Hugo Bello, Xoan Valledor, Jesús Pérez-González, Luis Ruiz-Valdepeñas; Efficacy of a Connected Insulin Pen Cap in People With Noncontrolled Type 1 Diabetes: A Multicenter Randomized Clinical Trial. Diabetes Care 2 January 2023; 46 (1): 206–208. https://doi.org/10.2337/dc22-0525
