# Supplementary figures and images for: Validation of a predictive calculator for optimal glycemic control and time-in-tight-range following CGM sensor placement in type 1 diabetes and pancreatic diabetes: a prospective study
Source: Endocrine. 2025 Aug 26;90(2):660–8. doi: 10.1007/s12020-025-04385-7 (PMC12572046; doi:10.1007/s12020-025-04385-7)

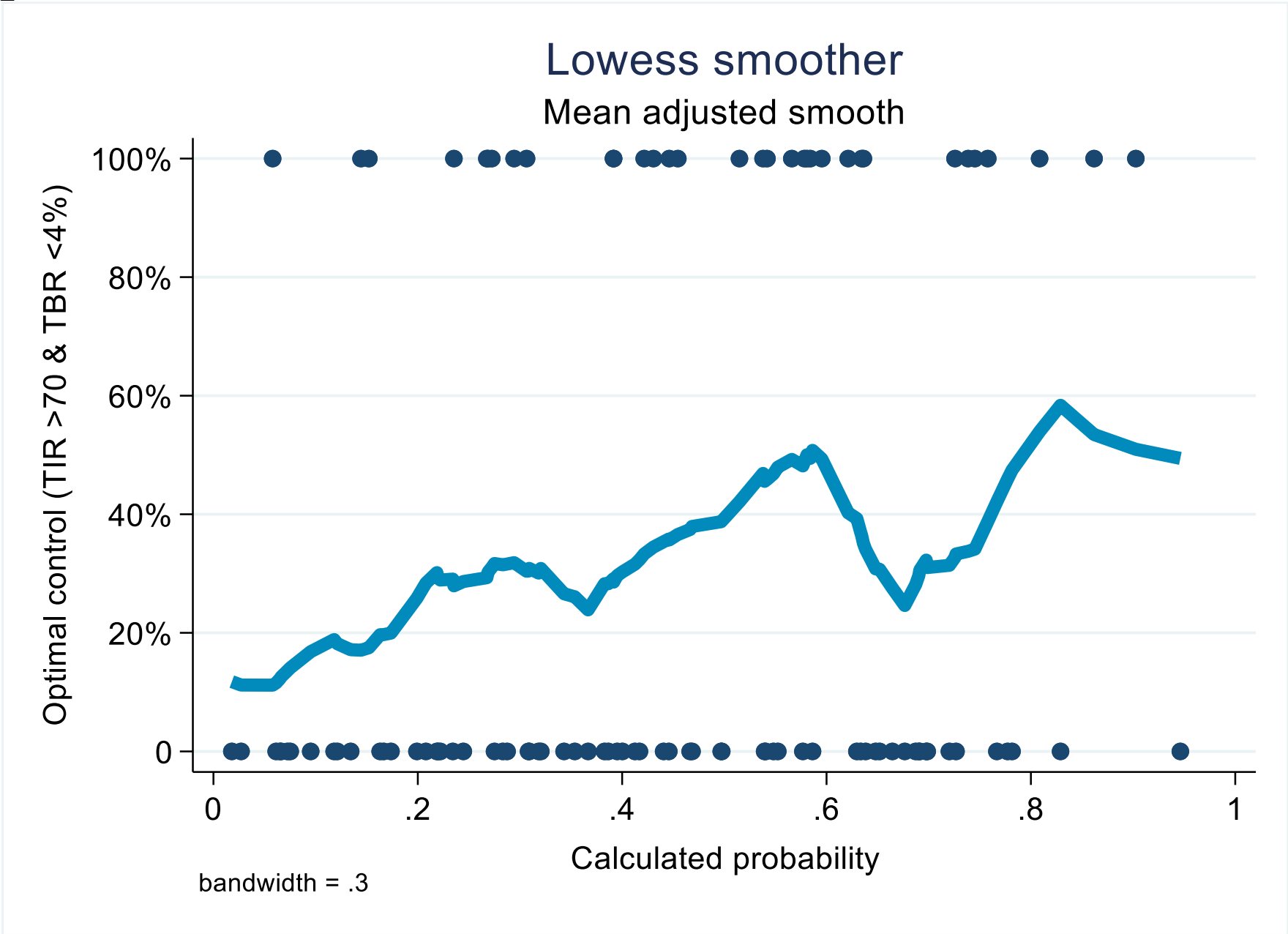

Supplement: Supplementary file 2 — Supplementary S2 [file 12020_2025_4385_MOESM2_ESM.pdf]
